# INCREASING SOCIAL CARE CAPACITY TO MEET THE NEEDS OF PERSONS AGING WITH DISABILITY

**DOI:** 10.1093/geroni/igac059.310

**Published:** 2022-12-20

**Authors:** Michelle Putnam, Lieke van Heumen, Lydia Odgen, Caitlyn Coyle, Kerri Morgan

**Affiliations:** Simmons University, Boston, Massachusetts, United States; University of Illinois at Chicago, Chicago, Illinois, United States; Simmons University, Boston, Massachusetts, United States; University of Massachusetts, Boston, Boston, Massachusetts, United States; Washington University School of Medicine in St. Louis, St. Louis, Missouri, United States

## Abstract

This national study presents key informant interview data (N=28) collected 2017-2018 identifying capacity building needs in professional skills, organizational operations, service/care models, and public policies to effectively serve older adults with long-term serious mental illness, intellectual/developmental disabilities, physical and sensory disabilities. A multi-university, interdisciplinary team collected data through phone interviews, coded, and analyzed transcripts thematically and using content analysis. Key informants worked in national affinity organizations, state-level organizations, academia, and social care organizations. Findings are broad in scope, highlighting variances in rural/urban areas, state government dispositions, workforce characteristics, social and health care infrastructure, beliefs about disability and chronic conditions, and other factors. Findings will informed the development of a larger survey study aiming to articulate an agenda for building capacity to meet the needs of aging with disability populations.

